# Architectural Refuges: Mapping Spatial Heterogeneity and Niche-Mediated Drug Resistance in Gastric and Esophageal Adenocarcinomas

**DOI:** 10.3390/cancers18111748

**Published:** 2026-05-27

**Authors:** Krishnapriya Thangaretnam, Md Obaidul Islam, Jialun Lv, Lei Chen, Farah Ballout, Shoumin Zhu, Heng Lu, Dunfa Peng, Wael El-Rifai, Zheng Chen

**Affiliations:** 1 Department of Surgery, Sylvester Comprehensive Cancer Center, University of Miami, Miami, FL 33136, USA; ktv17@med.miami.edu (K.T.); lxc1148@miami.edu (L.C.);; 2 Department of Veterans Affairs, Miami Healthcare System, Miami, FL 33125, USA

**Keywords:** spatial transcriptomics, gastroesophageal adenocarcinoma, tumor microenvironment, drug resistance, cancer-associated fibroblasts, spatial niche, metabolic reprogramming

## Abstract

Resistance to systemic therapy remains the defining challenge in the management of gastric cancer (GC) and esophageal adenocarcinoma (EAC). While genomic drivers of resistance are well characterized, traditional bulk profiling has failed to capture the physical rules governing tumor survival within the complex tissue ecosystem. Emerging data from 2024–2025, leveraging high-resolution spatial transcriptomics and multi-omics, have recontextualized resistance as a phenomenon of “spatial privilege” rather than solely an intrinsic cellular fate. This review summarizes recent evidence to define “architectural refuges”: distinct spatial niches that physically shield malignant clones from cytotoxic and targeted agents. We delineate three critical resistance domains common to upper gastrointestinal adenocarcinomas: (1) The “Excluded” Niche, where specific cancer-associated fibroblast (CAF) subpopulations (iCAFs vs. myCAFs) and stiffened extracellular matrix create hypovascular zones that limit drug delivery; (2) the “Immune-Tolerant” Niche, characterized by the spatial exclusion of CD8+ T cells and the recruitment of suppressive myeloid populations via the MIF/CD74 and USP14 axes; and (3) the “Metabolic” Niche, where mitochondrial heterogeneity and lipid metabolic symbiosis establish nutrient-deprived niches that select for stem-like, dormant states. By mapping these conserved spatial determinants from primary GEJ tumors to peritoneal and distant metastases, we argue that overcoming resistance requires an advancement: moving beyond targeting individual mutations to dismantling the multicellular architecture that sustains malignancy.

## Simple Summary

Gastric cancer and esophageal adenocarcinoma are exceptionally difficult to treat because cancers frequently develop resistance to therapies. Traditionally, scientists believed this resistance was primarily driven by genetic mutations inside the cancer cells. However, recent advances in high-resolution spatial mapping reveal that tumors actively construct complex physical and metabolic safe houses, termed architectural refuges, to shield themselves from drugs and immune cells. This review maps the three main types of these refuges and explains how they evolve dynamically as cancer spreads to other organs. By understanding how these physical barriers are built, researchers can design next-generation treatments that dismantle the protective tumor ecosystem rather than exclusively targeting individual cancer cells.

## 1. Introduction

Gastric cancer (GC) and esophageal adenocarcinoma (EAC) remain among the leading causes of cancer-related mortality worldwide, characterized by substantial molecular heterogeneity and a propensity for early metastasis. The recent paradigm shifts established by landmark clinical trials, such as CheckMate 649 and KEYNOTE 590, have solidified the role of combined cytotoxic chemotherapy (fluoropyrimidine and platinum) and PD-1 blockade as the frontline standard of care for advanced gastroesophageal adenocarcinomas. Furthermore, the incorporation of targeted agents against HER2 and Claudin 18.2 has expanded the precision oncology arsenal. However, despite these monumental clinical milestones, primary and acquired resistance remain ubiquitous. Current clinical stratification relies heavily on genomic signatures, such as microsatellite instability (MSI) and HER2 status [1,2]. Yet, even within genetically defined responders receiving these standard care regimens, clinical outcomes vary drastically [2]. This discrepancy suggests that resistance is not solely an intrinsic feature of the tumor cell genome but is dynamically enforced by the surrounding tumor microenvironment (TME).

For decades, our understanding of the TME was limited by “bulk” sequencing approaches, which average gene expression across tissue samples, effectively dissociating cells from their native context. This reductionist view obscures the physical rules of cell–cell interaction that govern tumor survival [3]. A malignant cell does not exist in isolation; its survival upon exposure to chemotherapy or immunotherapy is strongly influenced by its immediate “neighborhood”, the specific constellation of fibroblasts, myeloid cells, and vascular networks that physically shield it from therapeutic pressure [4].

The advent of high-resolution spatial transcriptomics and single-cell multi-omics in 2024–2025 has significantly advanced this paradigm from a “cellular” to a “spatial” perspective. We now recognize that drug resistance originates in specific “architectural refuges”: spatial niches where stromal remodeling creates immune-privileged or drug-inaccessible niches. For instance, recent spatial profiling has identified that resistance to fluoropyrimidine and platinum-based chemotherapy is often preceded by a specific remodeling of the extracellular matrix (ECM) and macrophage polarization in defined spatial zones, rather than global tissue changes [5,6]. Similarly, primary resistance to combined anti-PD-1 and anti-angiogenic therapy has been mapped to specific microenvironmental features that exclude functional T cells from the tumor core [7].

Furthermore, these spatial determinants are not static; they evolve dynamically from the primary tumor to metastatic sites. The architecture of a “permissive” niche in the stomach differs significantly from the “soil” required for colonization in the peritoneum or brain. Recent studies have elucidated unique evolutionary trajectories in which cancer cells hijack local stromal signals, such as the crosstalk between cancer-associated fibroblasts (CAFs) and macrophages, to construct immunosuppressive barriers in the peritoneum [8,9] or to induce atypical vasculature to survive in the brain [10].

In this review, we analyzed spatial omics data from 2024 and 2025 to define the “architectural refuges” of upper gastrointestinal adenocarcinomas. The rationale for a unified spatial framework for gastric cancer and esophageal adenocarcinoma stems from their shared biological identity as gastroesophageal adenocarcinomas (GEA). Extensive multi-omics profiling has demonstrated that esophageal adenocarcinomas strongly resemble the chromosomally unstable (CIN) variant of gastric adenocarcinoma, suggesting they should be considered a single disease entity driven by similar genomic instability and pronounced desmoplasia [11]. Consequently, while their anatomical origins differ, the ‘architectural refuges’ they construct—such as the exclusion of T cells by myofibroblastic fibroblasts—show remarkable conservation across the GEA spectrum. However, it is essential to note critically that current spatial omics platforms vary in their ability to resolve these niches, and much of the evidence remains correlational rather than functional [3]. While our primary focus is on the direct evidence derived from GC and EAC, the scarcity of EAC-specific spatial multi-omics requires a broader perspective. Therefore, where direct evidence is limited, we incorporate comparative and supporting evidence from esophageal squamous cell carcinoma (ESCC), colorectal cancer, and pan-cancer analyses. We explicitly qualify these supporting studies throughout the text, utilizing them to highlight conserved microenvironmental mechanisms, such as desmoplasia and immune exclusion, that are broadly applicable to gastrointestinal malignancies. We move beyond simple cell-type enumeration to describe functional spatial units: the “Excluded Niche” driven by fibroblast barriers [12], the “Immune-Tolerant Niche” orchestrated by myeloid checkpoints [13], and the “Metabolic Niche” shaped by nutrient gradients [14]. By mapping the geography of resistance, we provide a blueprint for next-generation therapies designed to dismantle the physical architecture of the tumor ecosystem.

To ensure a comprehensive and transparent review, we conducted a targeted literature search using PubMed, Web of Science, and Scopus databases. The primary search terms included combinations of (“spatial transcriptomics” OR “single-cell RNA sequencing” OR “multi-omics”) AND (“gastric cancer” OR “esophageal adenocarcinoma” OR “gastroesophageal junction”) coupled with (“tumor microenvironment”, “drug resistance”, “spatial niche”, or “cancer-associated fibroblasts”). Our selection criteria prioritized cutting-edge spatial and single-cell studies published between January 2024 and mid-2025, reflecting the recent technological explosion in high-dimensional tissue mapping. However, we also systematically included highly relevant foundational studies from earlier years (e.g., 2022–2023) if they provided critical mechanistic insights into therapy-induced TME remodeling or validated specific resistance pathways. Studies were selected for their ability to provide direct, high-resolution evidence of spatial architectures or cellular neighborhoods that influence tumor progression and treatment outcomes.

## 2. The “Excluded” Niche: Physical Barriers and Fibrotic Shields

In gastric and esophageal adenocarcinomas, resistance is frequently a problem of access rather than of potency [15]. The “Excluded” Niche is a spatial domain in which the tumor core is physically walled off from cytotoxic drugs and immune effector cells by a dense, desmoplastic stroma. Recent spatial transcriptomic analyses have moved beyond characterizing this fibrosis as mere scar tissue, identifying it instead as an active, biologically regulated fibrotic barrier constructed by specific subsets of cancer-associated fibroblasts (CAFs) and aberrant vascular units [12].

### 2.1. Spatial Polarization of CAF Lineages: ICAFs vs. myCAFs

The simplified view of CAFs as a monolithic population has been overturned by high-resolution mapping, which reveals distinct spatial lineages with opposing functions. Zhang et al. [12] utilized spatial transcriptomics to demonstrate that CAFs in gastric cancer are not randomly distributed but are organized into functional zones. They identified that inflammatory CAFs (iCAFs) are spatially anchored to cancer stem cells (CSCs), forming a dedicated “niche” that maintains stemness via paracrine signaling (e.g., WNT/EGFR pathways). In contrast, myofibroblastic CAFs (myCAFs) are predominantly located at the invasive margin, where they construct the dense collagen network that physically excludes T cells. This spatial dichotomy explains the failure of broad anti-fibrotic therapies; effective treatment requires dismantling the specific iCAF-CSC spatial unit rather than depleting fibroblasts globally. Furthermore, Zhou et al. [16] and Peng et al. [17] independently derived CAF-specific gene signatures associated with immunotherapy non-response, confirming that the qualitative state of the fibroblast neighborhood, specifically the enrichment of antigen-presenting CAFs (apCAFs), is a superior predictor of resistance than total stromal content. Despite the identification of these CAF subpopulations across multiple spatial datasets, the lack of standardized markers across different spatial transcriptomics platforms remains a significant hurdle. Many identified signatures are platform-dependent and have yet to be rigorously validated in prospective clinical trials to confirm their role as definitive drivers of resistance.

### 2.2. The Matrix as a Mechanical Signal: The Collagen/Cd44 Axis

The extracellular matrix (ECM) within the Excluded Niche functions not only as a physical barrier to drug diffusion but also as a mechanical signaling hub. Yang et al. [18] mapped a critical “Collagen/CD44 axis,” showing that stiff, collagen-rich niches mechanically activate CD44 signaling on tumor cells. This mechanotransduction directly induces stem-like properties and resistance to anoikis (cell death induced by detachment), effectively rendering tumor cells indifferent to cytotoxic stress. The clinical relevance of this mechanism is underscored by Dong et al. [15], who showed through combined bulk and single-cell analysis that beneficial responses to 5-fluorouracil (5-FU) are strictly correlated with an “ECM-depleted” phenotype, whereas non-responders exhibit a dense, cross-linked matrix that sequesters chemotherapeutic agents. Additionally, specific matricellular proteins like Periostin (POSTN), exclusively expressed by CAFs, have been identified by To et al. [19] as key drivers of this unfavorable, exclusionary architecture.

### 2.3. Chemoresistance Drivers: IGF1+ CAFs and Pericyte Barriers

Specific molecular drivers within the Excluded Niche have now been isolated as direct mediators of chemoresistance. Jia et al. [20] identified a distinct subpopulation of IGF1+ CAFs that protects adjacent gastric cancer cells from chemotherapy-induced apoptosis by activating survival pathways. This suggests that the “Excluded” niche provides a paracrine safety net during treatment. Finally, the vascular integrity of this niche is reinforced by pericytes. As comparative evidence, in esophageal squamous cell carcinoma (ESCC), a malignancy that shares intense desmoplastic features and vascular remodeling patterns with EAC, Pei et al. [21] discovered a subset of GPR116+ pericytes that tightly wrap tumor vessels. These pericytes do not normalize vessels for drug delivery; instead, they maintain a “leaky yet exclusive” vascular phenotype that promotes metastasis while restricting the extravasation of therapeutic antibodies. This is complemented by findings from Zhang et al. [22], who highlighted the fundamental transcriptomic divergence between tumor endothelial cells and their normal counterparts, further contributing to the immune-excluded state.

## 3. The Immune-Tolerant Niche: Hijacking the Defense

While the Excluded niche relies on physical barriers, the Immune-Tolerant niche employs spatial strategies to functionally paralyze infiltrating T cells. Recent spatial data suggest that this immunosuppression is not diffuse but highly structured, primarily driven by specialized myeloid networks and spatially restricted lymphoid aggregates.

### 3.1. Dominant Myeloid Checkpoints and Macrophage Barriers

Rather than a monolithic population, single-cell mapping reveals that specific tumor-associated macrophage (TAM) subsets serve as the primary architects of immune evasion. A dense macrophage barrier, characterized by SPP1-positive and C1QC-positive TAMs, actively excludes CD8-positive T cells [23]. The functional paralysis within this niche is enforced by dominant myeloid checkpoints. For example, the interaction between Siglec 10 on TAMs and CD24 on tumor cells serves as a powerful spatial “Don’t Eat Me” signal that substantially impairs phagocytosis, presenting a more actionable and dominant target than the classical PD-1 axis alone [13]. Secondary signaling cascades, such as the MIF/CD74 metabolic axis [24] and the USP14/IMP2/CXCL2 recruitment pathway [25], further stabilize this suppressive network by locking TAMs into a lipid-rich state and continuously recruiting myeloid-derived suppressor cells (MDSCs). Additionally, distinct subsets of exhausted T cells, marked by NKG2A expression, often co-occur with these myeloid barriers, necessitating multi-faceted reactivation strategies [26].

### 3.2. Spatial Organization Dictates Lymphoid Function

The concept of immune tolerance extends to the structural organization of lymphocytes. Spatial transcriptomics has elucidated a tertiary lymphoid structure (TLS) paradox, demonstrating that the functional capacity of TLS depends strictly on its spatial geography. Intratumoral TLS fosters active anti-tumor immunity and is enriched with mature dendritic cells, whereas peritumoral TLS is often functionally immature, trapped behind fibrotic barriers, and populated by immunosuppressive B cell phenotypes [8,27]. Thus, evaluating the spatial confinement and structural maturity of these aggregates is far more critical for predicting clinical response than merely quantifying total B cell infiltration.

## 4. The “Metabolic” Niche: Surviving Starvation

Beyond immune evasion and physical exclusion, the tumor microenvironment (TME) exerts profound selective pressure through metabolic heterogeneity. The 2024–2025 spatial multi-omics studies have revealed that drug resistance is frequently driven by “Metabolic Niches”—localized zones of nutrient deprivation or metabolic symbiosis that force cancer cells into a dormant, stem-like, or drug-tolerant state.

### 4.1. Spatial Metabolic Reprogramming and Lipid Symbiosis

Traditional bulk metabolomics fails to resolve where metabolic shifts occur. Using spatially resolved metabolomics and lipidomics, Sun et al. [14] constructed a high-definition metabolic map of gastric cancer. They discovered that metabolic reprogramming is not uniform but spatially compartmentalized. Specifically, they identified a distinct “tumor-normal interface” dominated by specific lipid profiles and immune cell infiltration, whereas the tumor core exhibited a profound accumulation of distinct signaling lipids.

This spatial lipid heterogeneity is functionally critical for metastasis. Yang et al. [28] elucidated a mechanism of stearoyl metabolism driving liver metastasis. They found that metastatic cells in the liver utilize specific fatty acid metabolic pathways to resist ferroptosis (iron-dependent cell death). The upregulation of NCOA4-mediated ferritinophagy in these niches maintains iron homeostasis, allowing cancer cells to survive in the lipid-rich hepatic microenvironment. This suggests that the “metabolic soil” at the metastatic site dictates the seed’s survival strategy.

### 4.2. Mitochondrial Defects and Glycolytic Dependencies

The metabolic niche is also defined by the functional status of mitochondria. Chu et al. [29] performed a comprehensive multi-omics analysis identifying that mitochondrial gene defects are not random but are key drivers of progression in specific patient subsets. These defects force tumor cells to rely on alternative survival pathways, creating specific vulnerabilities. Complementing this, Xu et al. [30] identified LDHA (Lactate Dehydrogenase A) as a top-ranking marker in malignant epithelial trajectories. The spatial enrichment of LDHA+ cells correlates with hypoxic zones where chemotherapy efficacy is compromised due to pH alterations and lack of oxygen radicals required for drug activity. Furthermore, Sun et al. [31] uncovered an ELK4-mediated mechanism in NDUFAB1+ tumor cells. This axis drives a dual program of metabolic reprogramming and immune evasion, directly linking mitochondrial function to the exclusion of cytotoxic T cells.

### 4.3. The Angiocrine Metabolic Support: NAMPT/ITGA5 Axis

Blood vessels in the TME are often viewed solely as transport conduits. However, Sung et al. [32] expanded this view by identifying endothelial cells as active metabolic “feeders” within the stem cell niche. Through spatially resolved transcriptomics, they demonstrated that deep-region endothelial cells secrete VISFATIN (extracellular NAMPT). This cytokine acts via the ITGA5-ITGB1 integrin receptor on adjacent cancer cells to boost NAD+ metabolism, thereby sustaining stemness and promoting resistance to immune checkpoint blockade. This defines a specific “angiocrine niche” where the vasculature itself provides the metabolic fuel for resistance, suggesting that targeting the NAMPT/ITGA5 axis could dismantle this protective niche.

To provide a comprehensive overview of the current landscape, Supplementary Table S1 summarizes the key spatial, single-cell, and multi-omics studies discussed in this review, highlighting their respective niche categories and clinical contexts.

**Figure 1:**
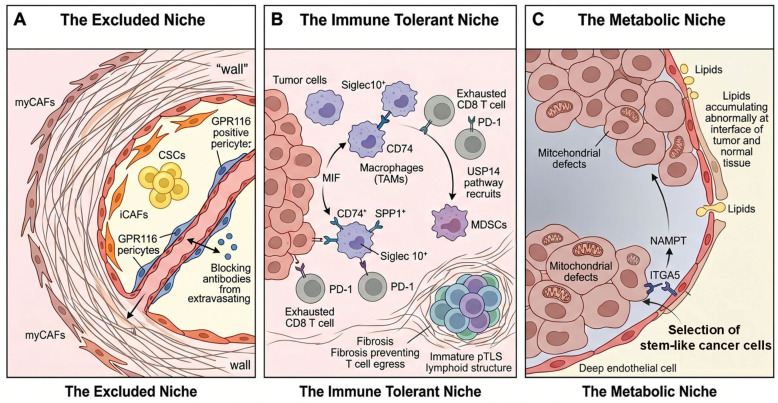
Schematic representation of the three architectural refuges mediating therapy resistance. (**A**) The Excluded Niche is characterized by a dense extracellular matrix forming a physical wall, showing the spatial segregation of inflammatory CAFs (iCAFs) near the tumor core and myofibroblastic CAFs (myCAFs) at the outer margin. It also depicts abnormal vascular coverage by GPR116-positive pericytes, which restricts therapeutic drug delivery. (**B**) The Immune-Tolerant Niche visually depicts the cellular and molecular interactions that drive immune exhaustion. Exhausted CD8+ T cells and immunosuppressive SPP1-positive macrophages are interacting directly with tumor cells via the MIF/CD74 axis. Additionally, the USP14 signaling pathway recruits myeloid-derived suppressor cells (MDSCs) and an immature peritumoral tertiary lymphoid structure (pTLS) physically enclosed by a fibrotic barrier. (**C**) The Metabolic Niche portrays a nutrient-deprived hypoxic core where cancer cells exhibit mitochondrial defects. It highlights the endothelial-derived NAMPT/ITGA5 signaling axis, providing metabolic support, along with lipid accumulation at the tumor margin, which together select for dormant, stem-like cancer cell populations.

A summary of the three functional spatial units is shown in Figure 1.

## 5. Spatial Evolution of Resistance: From Primary to Metastasis

A critical failure in current clinical practice is the treatment of metastatic disease based on the archival profile of the primary tumor. Spatial omics data from 2024–2025 provide evidence that the “resistance niche” evolves during dissemination. The architectural rules that protect a tumor cell in the stomach are deconstructed and rebuilt, often with higher fortification, in the lymph nodes, peritoneum, and distant organs.

### 5.1. The Lymph Node: A Remodeled Training Ground

Lymph nodes (LNs) are not merely passive filters but active sites of niche education. Hu et al. [33] utilized single-cell and spatial dissection to reveal a necroptosis-driven remodeling mechanism in gastric cancer LN metastases. Contrary to the view that necroptosis is immunogenic, they found that in the confined space of the LN, necroptotic signaling triggers a chronic inflammatory response that recruits immunosuppressive myeloid cells, effectively turning the LN from a zone of defense into a zone of tolerance. Furthermore, providing supporting evidence from a related tumor type, Guo et al. [34] mapped a specific MMP3 + IL24+ fibroblast subset enriched exclusively in metastatic LNs of esophageal squamous cell carcinoma (ESCC). Because ESCC shares critical lymphatic drainage pathways and nodal architecture with proximal EAC, these findings offer valuable mechanistic hypotheses for adenocarcinomas as well. These fibroblasts remodel the nodal architecture to support the survival of cycling, exhausted CD8+ T cells, preventing them from executing cytotoxic functions despite their expansion.

### 5.2. The Peritoneal Sanctuary: Fibrosis and Stemness

Peritoneal metastasis represents the most drug-resistant compartment in gastric and gastroesophageal cancers. Peng et al. [9] performed a definitive single-cell trajectory analysis comparing paired primary and peritoneal tumors. They found that peritoneal seeding is driven by a distinct differentiation trajectory in which cancer cells acquire a “mesenchymal-stem-like” phenotype largely absent in the primary tumor. This phenotype is sustained by a unique stromal niche characterized by high TGF-β signaling. The clinical implication of this niche was highlighted by the translational study of the PIPS-GC trial by Seo et al. [35], which identified specific molecular signals associated with resistance to intraperitoneal paclitaxel. They found that the peritoneal niche is less dependent on vascular delivery (which explains systemic chemo-failure) and more dependent on direct metabolic scavenging of peritoneal fluid. This creates a “pharmacologically privileged niche” where only agents capable of penetrating the dense, fibrous peritoneal stroma can succeed.

### 5.3. Distant Organs: Vascular Co-Option and Metabolic Adaptation

When gastric cancer cells colonize the brain or liver, they must overcome extreme environmental hostility. Liu et al. [10] provided the first spatial transcriptomic atlas of gastric cancer brain metastasis. They discovered that instead of inducing classical angiogenesis, metastatic cells in the brain adopt “atypical vasculature strategies,” mimicking endothelial cells (vasculogenic mimicry) to tap into the blood–brain barrier’s nutrient supply without recruiting immune cells. This “atypical vascular adaptation” renders anti-angiogenic therapies ineffective in the CNS. In the liver, the challenge is metabolic. Yang et al. [28] showed that liver metastases rely on a stearoyl-CoA desaturase (SCD)-mediated ferroptosis defense. The hepatic niche is rich in iron and lipids; to survive, metastatic cells upregulate NCOA4 to fine-tune ferritinophagy, preventing iron overload-induced death. This distinct metabolic dependency suggests that liver metastases might be vulnerable to ferroptosis inducers, a target irrelevant to the primary gastric tumor.

**Figure 2:**
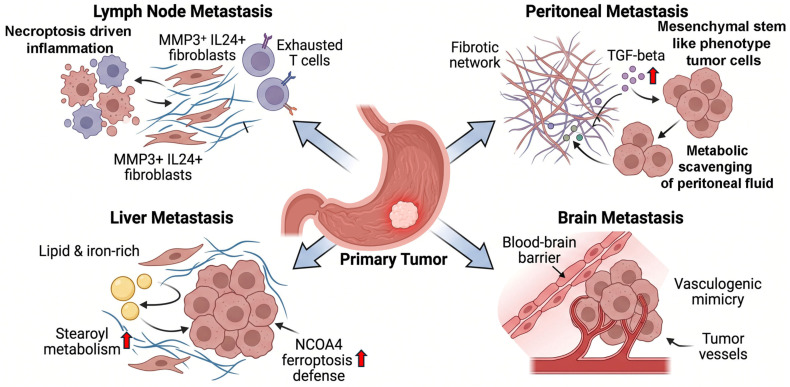
Spatial evolution of resistance niches during metastatic dissemination. The architectural rules that protect cancer cells at the primary gastroesophageal site are dynamically remodeled in distant organs. In the lymph nodes, necroptotic signaling and specific fibroblast subsets create a zone of tolerance. Peritoneal seeding is accompanied by a transition to a mesenchymal stem-like phenotype and reliance on direct metabolic scavenging. Hepatic colonization requires robust ferroptosis defense mechanisms mediated by stearoyl metabolism. Conversely, brain metastases evade therapy by adopting atypical vasculature strategies to breach the blood–brain barrier without relying on classical angiogenesis. Red arrows indicate up-regulation. Different colors’ circles show distinct cytokines.

A summary of the resistance niches is shown in Figure 2.

## 6. Clinical Implications and Future Directions

The transition from Section 2, Section 3, Section 4 and Section 5 illustrates a clear biological progression: tumors establish resistance by building physical barriers (the Excluded Niche), paralyzing immune responses (the Immune-Tolerant Niche), adapting to nutrient deprivation (the Metabolic Niche), and evolving these structures during metastasis. To provide a stronger foundation for these translational arguments, it is essential to contextualize these spatial discoveries against established clinical landmarks. Current standard-of-care regimens rely heavily on the systemic delivery of bulky cytotoxic agents and large monoclonal antibodies. Therefore, the architectural refuges described in this review represent the direct physical and biological reasons why regimens like CheckMate 649 ultimately fail in the majority of patients. Consequently, advancing clinical oncology requires an advancement beyond exclusively targeting single cellular mutations. Future strategies must focus on dismantling the specific physical and metabolic environments that restrict standard-of-care drug delivery and immune-cell penetrance. To directly translate our proposed spatial framework into practice, we outline a spatial stratification approach categorized into spatial diagnostics, niche-targeted therapies, and metastasis-directed interventions.

### 6.1. Spatial Diagnostic Approaches and Emerging Biomarkers

Predictive Spatial Phenotyping: As demonstrated by Wang et al. [27], the mere presence of B cells is insufficient for predicting immunotherapy response; pathology workflows may eventually need to quantify Intratumoral TLS (ITLS) versus Peritumoral TLS (pTLS). Similarly, Wang et al. [36] developed a spatial tool (‘Gastric-Discovery’) demonstrating that the specific spatial co-localization of ACTA2+ myCAFs and DAB2+ TAMs creates an immunosuppressive armor correlated with poor prognosis.

Early Detection and Epigenetic Biomarkers: Spatial profiling is also refining early detection. Huang et al. [37] used multi-omics to develop a 26-gene panel that spatially localizes high-risk intestinal metaplasia and distinguishes lesions likely to progress to GC from benign states. Furthermore, Sundar et al. [38] identified Alternative Promoter Burden (APB) as a critical epigenetic marker: GC cells that utilize alternative promoters generate truncated, non-immunogenic proteins, thereby establishing an immunologically “cold” spatial phenotype resistant to checkpoint blockade.

AI-Driven Multi-Omic Models: Integrating these complex datasets into deep learning models enables patient stratification based on multifaceted parameters [39,40]. Emerging prognostic signatures, such as CAF-related profiles [16], anoikis-regulating gene panels [41], and neutrophil-centric infiltration scores [42] (which also links to spatial microbiota composition), highlight the potential for AI to transform these varied microenvironmental signals into actionable clinical scores.

### 6.2. Niche-Specific Therapeutic Strategies

It is critical to note that the majority of the niche-specific targets discussed below (including Siglec 10, the NAMPT/ITGA5 axis, and ferroptosis modulation) remain strictly in the preclinical and exploratory phases. While these targets present strong mechanistic rationales, their clinical utility remains unproven and requires rigorous prospective validation before they can be considered viable therapeutic alternatives to established regimens. To translate these spatial insights into clinical practice, therapeutic strategies must prioritize targeting the foundational nodes of each niche rather than attempting to inhibit every aberrant molecular pathway. The objective is to systematically dismantle the architectural refuge, thereby resensitizing the tumor ecosystem to standard cytotoxic and immunotherapies.

#### 6.2.1. Normalizing the Excluded Niche

For tumors defined by severe desmoplasia, the highest therapeutic priority is restoring physical access to the tumor core. Rather than utilizing broad stromal depletion, interventions should target the specific cellular interactions maintaining the barrier. Disrupting the specific iCAF and cancer stem cell paracrine loop [12], or neutralizing the leaky vascular barriers maintained by GPR116-positive pericytes [21], represents a targeted approach to stromal and vascular normalization. Additionally, blocking the CCL2/STAT3 crosstalk [43] may attenuate the broader desmoplastic reaction, ultimately increasing the penetrance of systemic drugs.

#### 6.2.2. Reprogramming the Immune-Tolerant Niche

In immune-infiltrated but functionally suppressed tumors, the therapeutic hierarchy should center on breaking the dominant myeloid shields. Dual blockade strategies, such as combining anti-PD-1 with SIGLEC 10 inhibitors, offer the most direct and promising route to reinvigorating macrophage-mediated clearance in TAM-rich environments [13]. As a secondary architectural approach, preventing the spatial aggregation of suppressive cells by targeting chemokine recruitment axes (such as the CCL28/CCR10 pathway [44] or CXCR4 [45]) can prevent the assembly of the immune-tolerant niche before it fully matures. Furthermore, directly targeting the MIF/CD74 axis with agents like milatuzumab may reverse the lipid metabolic suppression of existing TAMs [24].

#### 6.2.3. Exploiting the Metabolic Niche

Overcoming metabolic refuges requires targeting the specific adaptive pathways that cancer cells utilize to survive nutrient deprivation and cytotoxic stress. High-priority vulnerabilities include targeting terminal differentiation states in docetaxel-resistant clones (marked by FOS and IFI27) [44] or inhibiting the FAK/AKT/mTOR axis using adjuvant compounds like Saikosaponin D to reverse cisplatin resistance [45]. Additionally, disrupting the angiocrine metabolic support provided by the endothelial NAMPT/ITGA5 pathway [32] or utilizing agents like the JAK inhibitor TG 101,209 to target RNA methylation regulators [46] may deprive dormant cells of the critical survival signals they rely upon during standard neoadjuvant therapy. Furthermore, targeting KLF9 overexpression presents a unique vulnerability specifically within stroma-enriched, platinum-resistant subtypes [47].

**Figure 3:**
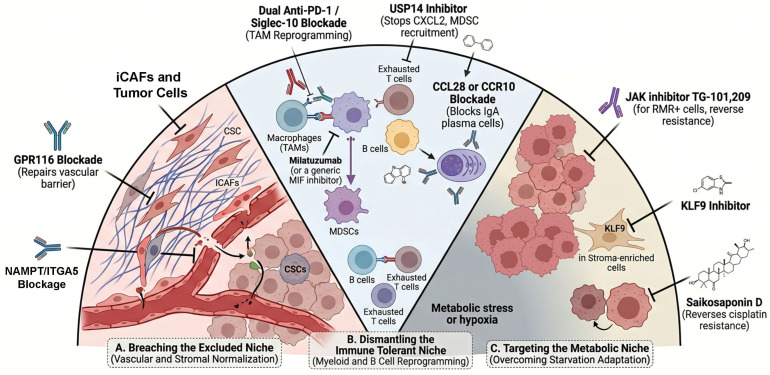
Therapeutic intervention map outlining targeted strategies to dismantle architectural refuges in gastroesophageal adenocarcinomas. The schematic is divided into three actionable zones corresponding to the spatial niches. In the Excluded Niche, treatments aim to normalize the stroma and vasculature by disrupting iCAF signaling, targeting GPR116-positive pericytes, and blocking the NAMPT/ITGA5 angiocrine loop. In the Immune-Tolerant Niche, therapeutic strategies focus on unlocking T cell cytotoxicity by reversing myeloid and stromal suppression, utilizing dual PD-1 and Siglec-10 blockade, MIF/CD74 inhibition, and targeting the CCL28/CCR10 or USP14 axes. In the Metabolic Niche, emerging interventions such as TG 101,209 and Saikosaponin D target metabolic plasticity and RNA methylation to sensitize tumor cells to conventional therapies. Arrows indicate transport or transformation.

To clarify the translational readiness of these emerging approaches, Supplementary Table S2 summarizes the key therapeutic targets discussed, explicitly distinguishing between exploratory spatial correlations, preclinical in vivo functional validation, and clinical associations. Figure 3 summarizes a therapeutic intervention map outlining targeted strategies.

### 6.3. Metastasis-Site-Specific Therapeutic Implications

As the resistance niche evolves during dissemination, therapeutic strategies must adapt to the metastatic site. In peritoneal metastasis, the niche is less dependent on vascular delivery and relies heavily on direct metabolic scavenging from peritoneal fluid, forming a fibrotic barrier [9]. The PIPS-GC clinical translational research demonstrated that responses to intraperitoneal paclitaxel depend on penetrating this unique stromal architecture, suggesting that local, penetration-enhanced delivery systems are required [35]. For lymph node metastases, preventing necroptosis-driven chronic inflammation may limit the recruitment of immunosuppressive myeloid cells [33]. Finally, for liver metastases, the unique dependency on stearoyl-CoA desaturase (SCD)-mediated defense against iron overload suggests that leveraging ferroptosis inducers could exploit the lipid-rich hepatic niche [28].

### 6.4. Limitations and the Challenge of Causation

While spatial transcriptomics and multi-omics provide unprecedented resolution, several critical limitations must be addressed to transition from descriptive atlases to interventional science. A major limitation of the current literature is the reliance on spatial correlation rather than functional causation. Co-localization of specific cell types (such as exhausted T cells and SPP1-positive macrophages) strongly implies interaction, but it does not definitively prove a causative mechanism of resistance. Furthermore, the field must reconcile conflicting findings across different patient cohorts. For instance, the TLS paradox demonstrates that the mere presence of tertiary lymphoid structures can correlate with both favorable and unfavorable outcomes depending on their spatial maturity and structural confinement. Validating these complex, context-dependent spatial signatures requires robust functional models and large-scale, multi-center prospective cohorts to ensure these specific niche architectures are broadly reproducible and truly drive clinical outcomes.

### 6.5. Operational Clinical Translation and Pathology Workflows

The transition from descriptive spatial atlases to precision spatial medicine requires overcoming significant operational hurdles. Currently, clinical oncology relies heavily on established, single-analyte bulk biomarkers such as HER2 amplification, microsatellite instability (MSI), and PD-L1 Combined Positive Score (CPS). While these standard markers are highly useful for rapid clinical decision-making, they often fail to capture the spatial context that dictates therapy resistance.

To realistically integrate spatial stratification into current pathology workflows, the field cannot rely on whole transcriptome spatial profiling, which remains prohibitively expensive and computationally demanding. Instead, discovery platforms must identify minimal, highly predictive biomarker sets that can be translated into targeted multiplex immunohistochemistry (mIHC) or multiplex immunofluorescence (mIF) panels. These targeted panels must be optimized for standard formalin-fixed, paraffin-embedded (FFPE) archival tissues and adhere to Clinical Laboratory Improvement Amendments (CLIA) standards. Overcoming implementation barriers requires rigorous assay standardization, establishing inter-platform reproducibility, and developing automated clinical decision algorithms that can deliver actionable prognostic scores within the standard turnaround time required for first-line treatment initiation.

#### 6.5.1. Assay Standardization and CLIA Integration

While whole-transcriptome spatial profiling remains discovery-oriented, translating these multi-omic signatures into targeted multiplex immunofluorescence (mIF) or multiplex immunohistochemistry (mIHC) panels is essential for integration into Clinical Laboratory Improvement Amendments (CLIA)-certified laboratories. This transition requires rigorous standardization of pre-analytical variables, such as tissue fixation times, ischemia times, and sectioning protocols, particularly since these assays must perform reliably on standard archival formalin-fixed, paraffin-embedded (FFPE) tissues. Furthermore, establishing inter-platform reproducibility and developing standardized clinical decision algorithms are necessary to ensure that spatial scores can reliably guide treatment choices across different institutions without subjective pathologist bias.

#### 6.5.2. Workflow Realities and Turnaround Time

For successful clinical translation, diagnostic assays must meet the rapid turnaround time demands of advanced gastroesophageal cancers. Current high-plex spatial platforms require several days to weeks for data acquisition and complex computational analysis, which is incompatible with making urgent first-line treatment decisions. Future companion diagnostics (CDx) derived from spatial data must be streamlined to deliver actionable results within the standard five to seven-day pathology workflow.

#### 6.5.3. Regulatory Barriers and Reimbursement

The regulatory approval of complex spatial signatures as companion diagnostics poses a unique challenge. Agencies like the FDA require extensive analytical and clinical cross-validation to ensure safety and efficacy. Furthermore, for widespread clinical adoption, assay developers must demonstrate the clinical utility and cost-effectiveness of these advanced diagnostics to secure dedicated reimbursement codes from healthcare payers and insurance networks. Without clear reimbursement realities, even the most predictive spatial assay will fail to reach the clinic.

#### 6.5.4. Prospective Validation Design

Finally, the current literature relies almost entirely on retrospective cohorts. Future efforts must prioritize prospective clinical trial designs that use validated spatial biomarkers as integral patient-stratification factors. These trials must move beyond mere observation to interventional designs, determining whether targeting specific cellular neighborhoods, such as reprogramming tumor-associated macrophages or disrupting specific cancer-associated fibroblast niches, provides a statistically significant survival benefit when combined with standard chemotherapy and immune checkpoint blockades.

## 7. Conclusions

The resistance of gastric and esophageal adenocarcinomas to systemic therapy is not merely a consequence of genomic instability but is actively engineered by the spatial architecture of the tumor microenvironment. The data emerging from 2024 and 2025 strongly suggest that resistance resides in specific “Architectural Refuges”: the excluded niche that walls off therapy, the immune-tolerant niche that paralyzes defense, and the metabolic niche that fuels survival under stress.

By moving from single-cell reductionism to spatial reconstruction, we have begun to map the spatial progression of resistance in current treatments. The future of GI cancer therapy lies in precision spatial medicine: strategies designed not just to kill cancer cells, but to dismantle the physical and metabolic niches that protect them. Whether through normalizing the stroma, reprogramming the myeloid barrier, or targeting the metabolic soil of metastatic sites, the next future therapeutic advances may depend on treating the tumor ecosystem as a structured, spatially organized organ.

## Data Availability

No new data were created or analyzed in this study. Data sharing is not applicable to this article.

## Supplementary Materials

The following supporting information can be downloaded at: https://www.mdpi.com/article/10.3390/cancers18111748/s1, Supplementary Table S1. Summary of key spatial, single-cell, and multi-omics studies defining architectural refuges in upper gastrointestinal cancers. Supplementary Table S2. Summary of therapeutic targets in gastroesophageal adenocarcinomas and their respective levels of evidence.
